# How clinical reasoning is taught and learned: Cultural perspectives from the University of Melbourne and Universitas Indonesia

**DOI:** 10.1186/s12909-016-0709-y

**Published:** 2016-07-21

**Authors:** Ardi Findyartini, Lesleyanne Hawthorne, Geoff McColl, Neville Chiavaroli

**Affiliations:** Department of Medical Education, Faculty of Medicine, Universitas Indonesia, Jakarta, Indonesia; Melbourne School of Population and Global Health, University of Melbourne, Melbourne, Australia; Melbourne Medical School, Faculty of Medicine Dentistry and Health Sciences, University of Melbourne, Melbourne, Australia

**Keywords:** Clinical reasoning, Undergraduate medical education, Cultures of learning, Comparative case study

## Abstract

**Background:**

The majority of schools in the Asia-Pacific region have adopted medical curricula based on western pedagogy. However to date there has been minimal exploration of the influence of the culture of learning on the teaching and learning process. This paper explores this issue in relation to clinical reasoning.

**Method:**

A comparative case study was conducted in 2 medical schools in Australia (University of Melbourne) and Asia (Universitas Indonesia). It involved assessment of medical students’ attitudes to clinical reasoning through administration of the Diagnostic Thinking Inventory (DTI), followed by qualitative interviews which explored related cultural issues. A total of 11 student focus group discussions (45 students) and 24 individual medical teacher interviews were conducted, followed by thematic analysis.

**Results:**

Students from Universitas Indonesia were found to score lower on the Flexibility in Thinking subscale of the DTI. Qualitative data analysis based on Hofstede’s theoretical constructs concerning the culture of learning also highlighted clear differences in relation to attitudes to authority and uncertainty avoidance, with potential impacts on attitudes to teaching and learning of clinical reasoning in undergraduate medical education.

**Conclusions:**

Different attitudes to teaching and learning clinical reasoning reflecting western and Asian cultures of learning were identified in this study. The potential impact of cultural differences should be understood when planning how clinical reasoning can be best taught and learned in the changing global contexts of medical education, especially when the western medical education approach is implemented in Asian contexts.

## Background

The Asia-Pacific region has the largest population and the greatest number of medical schools in the world [1, 2]. The scale of medical school development in the past 2 decades has been apparent, with the number of recognised institutions rising from 883 to 1217 in the past decade alone [1, 2]. This is mirrored in Indonesia, where 19 new medical schools have opened in the past 2 years, with numbers increasing from around 40 to over 70 in the last decade [3]. This increase in scale and the simultaneous adoption of educational innovations such as problem based learning and early clinical training [4], has encouraged studies on the impact of importing western educational pedagogy into Asian cultural settings [5, 6].

In the past 2 decades a growing number of analysts have contended that Asian students bring profoundly different cultures and values to medical learning when compared to their western counterparts. These are significant in relation to overall approaches to learning [7, 8], motivation, the perceived value of student versus teacher authority [9], and critical thinking [10].

Clinical reasoning (CR), the focus of the current paper, is a complex and critical skill for medical graduates to develop. In the past 30 years, the western medical education literature has conceptualised it as a cognitive and reflective process drawing on doctors’ knowledge and clinical experience, leading to definition of the diagnosis and management plan [11, 12]. It was originally considered to be a generic skill that could be applied to any clinical problems [13, 14]; however research increasingly suggests that it is more usefully viewed as an interactive [12], context specific [15, 16], and multifactorial [17] process. Teaching and learning CR in the undergraduate medical program has been viewed as critically important [15]. Multiple strategies to develop this competency among medical students have been employed [18–20]. At the same time, given that CR skills involve relevant knowledge acquisition, clinical experience and thinking processes [15, 16], all strategies implemented in medical education may implicitly facilitate CR skills development.

Therefore, several cultural differences in relation to learning have potential significance for the acquisition of CR competencies given the widespread adoption of western pedagogies in Asian, African and Middle Eastern universities. It is thus worth examining the ways in which the cultural context of learning CR may influence the process and final outcomes of medical learning in general [21].

### Cultural dimensions based on Hofstede’s research

Based on a body of globally influential qualitative research conducted in 64 countries over more than 40 years, Hofstede and colleagues proposed 4 cultural dimensions of potential relevance to learning clinical reasoning [22, 23]. These dimensions were used as analytical frameworks in this study since they provided spectrums against which each country’s relative position could be identified [22, 23]. The dimensions are [22, 23]:

- *Power distance*. This refers to expectation of inequality and centralised authority in a large power distance culture as opposed to participative decision making and egalitarianism in a small power distance culture. In terms of education this translates to the perceived level of equality between teachers and students, including comparative levels of expertise. While medical education in most western countries is characterised by small power distance, including a trend towards student-centered learning [10], in most eastern countries with large power distance education remains a more traditional teacher-centered process [22]. Teachers as experts may provide examples of different CR strategies in solving clinical cases. On the other hand, CR skills still need to be processed and learned individually.
- *Uncertainty avoidance* (i.e. from weak to strong). This concerns the extent to which members of a culture adapt to or avoid uncertain and unknown situations. Cultures with strong uncertainty avoidance tend to seek predictability e.g. in relation to written and unwritten rules. Cultures with weak uncertainty avoidance, by contrast, are characterised by tolerance of differing behaviours and opinions, and greater flexibility [18]. Given the unpredictability of the clinical cases encountered in clinical settings, CR skills and its learning require the capacity to deal with uncertainty. A greater emphasis should therefore be placed on developing this capability in addition to an ability to utilise relevant knowledge for particular cases [24].
- *Collectivism* versus *individualism*. Collectivist cultures require people to be cohesively integrated in highly loyal groups. By contrast in individualistic cultures every person is expected to be reliable and look after him/herself. In higher education, this may be reflected in relation to different dynamics in small group activities in which students need to learn collaboratively while also assuring individual accountability. Prior studies have highlighted the learning challenges faced by Asian students from more collectivist cultures [25] when required to express their ideas in group tutorials and conduct self-directed learning [26, 27], in contrast to western students who are keen to learn more individually [28].
- *Masculinity* versus *femininity*. Finally, masculine cultures are characterised by clearly differentiated gender and social status roles, whereas feminine cultures embrace greater overlapping. In terms of education, in more feminine cultures, the ‘average’ student is considered the norm, whereas in more masculine cultures, students striving for the ‘best’ achievement are encouraged [22].

Educational studies based on Hofstede’s framework have been conducted in a range of tertiary settings [29–32]. Two by Jippes and Majoor specifically correlated these 4 dimensions to the implementation of medical curricula [31, 32]. The first highlighted the percentage of integrated and Problem Based Learning (PBL) curricula in 17 Europe countries (2008), while the second assessed this process in 64 countries representing all global continents (2011). A separate medical study explored cultural dimensions and their relation to feedback provision in clinical settings [33].

No research to date however has examined the impact of cultural differences on the acquisition of CR skills in different medical schools [11]. The current study addressed the following questions: a. To what extent is the teaching and learning of clinical reasoning conceptualised differently in different settings? and b. How do any differences in cultural values influence the teaching and learning of CR skills?

## Methods

A comparative case study design [34, 35] was adopted to explore the impact of cultural differences on CR in an Asian compared to western medical education setting in 2008–09, using quantitative and qualitative data. A case study examines select case(s) in order to understand and explore the specific phenomenon within defined contexts [34, 36] by obtaining data from multiple sources over a fixed period of time [34, 35]. The comparative case study design was adopted because it allows direct comparison by replicating a range of methods, and drawing on qualitative and/or quantitative approaches between cases in order to provide different perspectives of relevance to the research focus [34–36].

Potential differences in relation to CR teaching and learning were thus explored at 2 medical schools in the Asia-Pacific region. While one reflected Asian and the other western cultural and educational traditions, it is important to note that they shared certain similarities, including clear definition of CR skills as a major learning objective, adoption of PBL as a primary teaching-learning method, integration of biomedical and clinical knowledge across the curriculum, and implementation of early clinical exposure.

Despite these similarities, there were several key differences in the way the University of Melbourne (UoM) and Universitas Indonesia (UI) implemented the curriculum at the time the study was conducted. First, UoM admitted both undergraduate and graduate medical students, whereas UI admitted only undergraduate students. Second, UoM implemented a 5 semester clinical practice training with an integrated approach in the end, whereas UI conducted a 4 semester clinical practice training with departmental-based approach. Third, as will be demonstrated, there were significant cultural differences in the teaching and learning approach, including in relation to clinical reasoning.

The first stage of the study involved administering the Diagnostic Thinking Inventory (DTI) to students in semester 6–at the end of preclinical year and 12–at the end of clinical year. The DTI is a 41-item instrument designed to quantitatively measure diagnostic ability, in particular students’ degree of ‘flexibility in thinking’ and the ‘degree of knowledge structure in memory’ [37]. The DTI was further utilised because it measures the style and attitude of diagnostic reasoning, and does not involve assessment of actual knowledge application nor the results of clinical reasoning process (for example, diagnosis and treatment accuracy). DTI results reflect students’ level of expertise in diagnostic reasoning, as an important part of their overall clinical reasoning skills at a specific point in time. The first factor of DTI, ‘flexibility in thinking’, adopts both *deterministic enquiry* (where a hypothesis is first developed and then information gathering is processed based on memorised knowledge), and *responsive enquiry* (where a clinician follows up new information provided by the patient). The degree of flexibility in thinking should enable the physician to shift between these 2 modes [37]. A high score on ‘knowledge structure or organisation’ reflects an advanced level of elaborated and compiled knowledge [36] which is possessed by an effective diagnostician who will recognise and solve the clinical case as a whole.

The DTI was translated into Indonesian for administration at the UI, a process involving back translation into English, comparative analysis of the Indonesian and English versions, and independent verification by bilingual and qualified academics [38]. To supplement the data obtained through the DTI administration, medical teachers’ (*N* = 24) and medical students’ (*N* = 45) perceptions of CR teaching and learning in each medical school were subsequently explored through in depth individual interviews and focus group discussions (FGD).

### Research sample

#### Medical students

Medical students volunteering to participate in this study were enrolled in semesters 6 and 12 at the UoM and the UI. They were recruited through student emails or direct meetings with the researchers (in line with ethics approval). Given the implementation of PBL tutorials up to semester 6, and the exposure to clinical training of semester 12 students, the research was designed to capture any differences in students’ DTI scores, as well as any differing cultural perspectives in regards to the clinical reasoning and pedagogical strategies employed in the contrasting institutional settings. It was anticipated that a sample of 250 students for each cohort would be sufficient to detect any true difference in performance on the DTI (5 % alpha level, 80 % statistical power) [36]. Three to six FGDs were conducted at each university after its administration, with 3–6 participants from each semester recruited for each FGD. The FGD questions included students’ perceptions of CR, how they learned CR, and the current teaching approaches (Table 1).

**Table 1:** Questions for medical teacher interview and student FGD

A	MEDICAL TEACHER INTERVIEW QUESTIONS
	1. How long have you been involved in teaching medical students? In what activities have you been involved?
	2. How would you define clinical reasoning (in terms of medical students’ training)?
	3. Do you think that clinical reasoning can be taught to medical students? Would you please explain why/ why not?
	4. In your experience, what are the issues in teaching and learning clinical reasoning? How do you do the teaching well?
	5. What do you think about the overall medical curriculum you’re involved in? Do you think that it facilitates clinical reasoning teaching and learning? (If so, how?)
B	STUDENT FGD QUESTIONS
	GENERAL QUESTIONS (OPENING)
	1. When you’re given the term ‘clinical reasoning’, what comes into your mind/what picture that comes into your mind?
	2. How would you describe clinical reasoning to me as a person who knows nothing about clinical reasoning?
	KEY QUESTIONS
	3. What is the best way to learn clinical reasoning? Can you please give some examples?
	4. To what extent the medical course has been able to do this for you?
	5. Which learning activities in the course have been most useful to you in your learning of clinical reasoning? And which learning activities in the course have been less useful to you?
	6. What stage of the course do you think is the best time to learn clinical reasoning?
	FINISHING QUESTION
	7. All of Medical Education Unit academic staffs have run away, and you’re the only one left. How would you set it up?

#### Medical teachers

The study further sought information from medical teachers representing a range of teaching roles through semi-structured interviews: PBL tutors, basic clinical skills tutors, biomedical teachers, and clinical teachers. Informants were selected purposively according to their level of involvement in the design and/or delivery of the curriculum and were directly invited to participate. The interviews explored their understanding of clinical reasoning and the teaching and learning strategies employed in each institution (Table 1). Fourteen to sixteen interviewees were sought in each medical school, securing sufficient numbers across the range of teaching roles. The total number of interviews was determined by the preliminary results of interview data analysis, informed by data saturation [38].

#### Method of analysis

The internal consistency of DTI was assessed, and the DTI scores between semester 6 and 12 groups in each setting were compared using ANOVA and the Kruskal - Wallis tests, depending on data distributions. Nonparametric tests were used when the data distribution were not normal. Further item analysis was also completed to compare the consistency of responses to DTI item in both settings.

All FGD interviews were taped, transcribed verbatim, and thematically analysed. Hofstede’s 4 cultural dimensions [22, 23] were adopted as the framework for analysis, with the researcher adopting an open mind. Significant quotes were coded and categorised iteratively to develop a comprehensive set of themes [39]. Further comparative analyses of the UoM and the UI data were conducted to allow key themes related to cultural issues in CR teaching and learning emerged [39, 40]. Following an independent thematic verification of sample transcripts by the 3 authors (AF, LH, NC) to ensure coding reliability [41], one author (AF) completed manual data analyses.

This study was approved by the Research Ethics Committees at the Faculty of Medicine, Dentistry and Health Sciences (UoM) and the Faculty of Medicine (UI).

## Results

### Quantitative results

At the UoM, 69 semester 6 students and 97 semester 12 students, and at the UI 75 semester 6 and 128 12 students were recruited to the study. The demographic characteristics of these samples are shown in Table 2.

**Table 2:** Demographic characteristics of the University of Melbourne and the University of Indonesia samples

Characteristics	University of Melbourne				University of Indonesia			
	Semester 6 (*N* = 69)		Semester 12 (*N* =97)		Semester 6 (*N* = 75)		Semester 12 (*N* = 128)	
	n	%	n	%	n	%	n	%
*Gender*								
Male	31	44.9	34	35.1	30	40.0	59	46.1
Female	38	55.1	63	64.9	45	60.0	69	53.9
*Age group*								
16–20 yo	32	46.4			30	40.0	126	98.4
21–25 yo	37	53.6	60	61.9	45	60.0	2	1.6
26–30 yo			29	29.9				
31–35 yo			5	5.2				
More than 36 yo			3	3.1				

The analysis of internal consistency revealed that the DTI reliability coefficients were consistently high for both UoM and UI data (0.7–0.8). These results were comparable to the total score reliability coefficients obtained by Bordage et al. [37] and other studies which have utilised the DTI [42–44]. The major difference was the lower internal consistency of flexibility in thinking subscales at the UI (0.55), compared to the internal consistency at the UoM (0.79).

Further item analysis revealed that there were 4 problematic items in the DTI Flexibility subscale at the UI, which were not found to be problematic at the UoM (Flexibility items 2, 6, 13 and 18). This may be explained by the fact that UI students who had high scores in the DTI appeared to be ambivalent in deciding whether they needed to: a) think about the diagnostic possibilities early on in the case (Flexibility item 2), b) ask the patient to define the symptoms more clearly (Flexibility item 6), c) make a decision based on comparing and contrasting the various possible diagnoses (Flexibility item 13), and d) be prepared to change their mind about a patient (Flexibility item 18).

A comparison of the DTI score across the 4 sub-groups was conducted using non-parametric tests (Kruskal-Wallis and Mann–Whitney test), given that some data sets (namely DTI Flexibility in semester 6 UoM, and in semester 6 and 12 UI) did not have normal distributions (test of normality *p* < 0.05) (Table 3). Further evaluation using the Mann–Whitney test revealed that UoM and UI semester 12 respondents consistently had higher scores on the 2 DTI subscales and total DTI scores compared to their semester 6 counterparts (*p* < 0.0125; Bonferroni adjustment of p 0.05/4 Mann–Whitney tests across locations and semesters). On the other hand, there were no significant differences in the total DTI scores between the UoM and the UI in either semester 6 or semester 12 (*p* > 0.0125).

**Table 3:** DTI results at the University of Melbourne and Universitas Indonesia

Institution	Semester	DTI scores	N	Mean (range)^a^	*SD*
University of Melbourne	Semester 6	DTI knowledge in memory score (max = 120)	69	74.91 (55–102)	8.36
		DTI flexibility in thinking score (max = 126)		82.41 (64–105)	8.64
		Total DTI score (max = 246)		157.32 (119–207)	15.68
	Semester 12	DTI knowledge in memory score (max = 120)	97	82.84 (47–104)	10.20
		DTI flexibility in thinking score (max = 126)		86.89 (60–109)	10.26
		Total DTI score (max = 246)		169.76 (113–212)	19.32
Universitas Indonesia	Semester 6	DTI knowledge in memory score (max = 120)	75	72.45 (44–96)	10.5
		DTI flexibility in thinking score (max = 126)		79.47 (53–116)	9.0
		Total DTI score (max = 246)		151.97 (109–211)	17.34
	Semester 12	DTI knowledge in memory score (max = 120)	128	80.89 (59–106)	8.94
		DTI flexibility in thinking score (max = 126)		84.7 (61–107)	8.64
		Total DTI score (max = 246)		165.66 (121–212)	15.18

^a^Significant difference in the DTI memory, DTI flexibility and total DTI scores across the 4 groups (Kruskal-Wallis test, *p* < 0.001)

### Qualitative results

A total of 11 student FGDs were conducted: 3 in semester 6 with a total of 16 students and 4 in semester 12 (15 students) at the UI, and 2 in semester 6 (7 students) and 2 in semester 12 (7 students) at the UoM. As noted, there were slight differences among the FGDs in these 2 institutions related to the admission pathway. UoM participants comprised high school leavers (12) and graduate-entry students (2), whereas UI students were all high school leavers (31). UoM also included the presence of international students: 6 in the focus group sample, largely derived from South East Asia, compared to 8 domestic students. Some had been in Australia a substantial period of time.

A total of 24 interviews were also completed with academics (11 at the UI, 13 at the UoM), with equal gender representation and a comparable level of seniority (at least 7 years’ experience). Given these equivalent characteristics, any differences in views were expected to be teaching role-specific, either as PBL tutors (*n* = 3), basic clinical skills tutors and clinical teachers (hospital based-medicine, *n* = 3, hospital based-surgery, *n* = 3 and general practice based, *n* = 5) at the UoM and either as PBL tutors (*n* = 3), basic clinical skills tutors and clinical teachers (hospital based-medicine, *n* = 3, hospital based-surgery = 3 and general practice based, *n* = 2) at the UI. Alternatively, they could have been influenced by culture. Data saturation was evaluated based on the teaching roles in each setting and was reached adequately.

Two major themes emerged in relation to the potential impact of culture of learning on the teaching and learning of CR: power distance (attitude to authority), and uncertainty avoidance, as described below.

- Power distance
- The perception of academics on content expertise and the process of knowledge transfer
  While academics from both universities considered knowledge to be essential to effective clinical reasoning, UI academics were far more likely to emphasise the importance of their content expertise. For instance, 1 view relating to the significance of pharmacology in clinical reasoning stated:
  “In the end, they only discuss the clinical [aspects] and [they do] not [discuss] them comprehensively. There was no opportunity [to discuss] pharmacology and yet it is actually pharmacology that connects basic science to clinical studies, …… Pharmacology is the bridge in my opinion.” (PBL tutor 1 UI).
- Reliance on teachers’ role as a source of information
  Student reliance on teacher expertise was reported by students at both institutions. However UI students placed far greater reliance on teachers as the expert source of information, and as facilitators to guide tutorial discussion. For example:
  “…the standard of the tutors in applying the system is different from 1 tutor to another. Therefore, we got different knowledge if we compare it to other groups. That’s also a problem, I think. But, I think if the tutors themselves apply the same standard to lead us in the discussion, and have standard of information to be given to students, I think there will be more balance to it. Balance, I mean, that the tutor has to give clear direction during the discussion.” (FGD semester 6 UI 1)
  By contrast, UoM students were clearly accustomed to a culture with a small power distance. They appeared more independent in their learning and tended to regard teachers as facilitators rather than content experts, as illustrated by the following comment:
  “…There’s a tutor who only sits back and just watches us, and there are teachers who follow the discussion, and there’s also a tutor who tells you everything.. [Having a tutor between the 2] gives students some freedom.” (FGD semester 6 UoM 1)
- *Students*’ versus *teachers*’ *views on the importance of patient collaboration in the clinical reasoning process*.
  Cultural difference in attitudes to authority in these 2 settings was also revealed in the perceived importance of patient collaboration in the clinical reasoning process. UoM academics valued patient collaboration and input as significant in the clinical reasoning process. For instance, as stated by 1 of the clinical teachers, the way a patient describes and perceives breathlessness may vary between patients, and this will influence the doctor’s clinical reasoning process. By contrast, this issue was rarely considered by UI academics, and seemed irrelevant to UI students’ views. The patient was not seen to have experiential authority in relation to clinical conditions.
- Capacity to deal with uncertainty
- The need for adequate knowledge to start the clinical reasoning process
  Despite semester 6 UI students’ expectations that they learn CR through PBL, they were unsure if knowledge and clinical reasoning could be learned side-by-side, as illustrated below:
  “The [PBL tutorial] trigger we’ve been having, [in order] to learn the case is still limited. Because a lot of them [who] aim to encourage us to have self-directed learning, and explore by ourselves. To learn clinical reasoning, what we need is the real case. For example, we find these symptoms, and from the physical examination we have these, and when we ask for lab investigations, we are provided with the results, and from there, we may be able to explain why we [decide] to do certain actions….” (FGD semester 6 UI 2)
  The PBL tutors at UI were also unsure that PBL tutorials could be used to teach CR skills, as reflected by the following comment:
  “We [tutors] have to emphasise that [a clinical] interest is not inappropriate; however, they need to explore the basics first. That’s the aim at this medical science stage. Later on, when they arrive at the clinical years, they will have the opportunity to apply their knowledge to the real patients. That’s where clinical reasoning is really trained.” (PBL tutor, UI)
  This was in contrast with UoM teachers and students, who agreed that while CR required knowledge, they had sufficient confidence to proceed in PBL on a basis of limited knowledge. By way of example:
  “I still find it amazing when on the first day of PBL …we really did not have any medical knowledge. I think it was the abdominal pain and diarrhoea [case]. And you know, ten people in the same room, just from common knowledge from high schools and from day to day life, could come up with quite reasonable hypotheses…..” (FGD semester 12 UoM 2).
  A UoM teacher supported this view:
  “Again early on, it’s what PBL is clearly doing, and that’s great, a little bit about diagnostic reasoning, and that’s great, and see the relevance of the physiology, anatomy, etc. in their learning. But the bulk of it still needs to come later. They need the knowledge.”
  (Clinical teacher 1, UoM)
- Introduction of pattern recognition strategy
  UI academics rarely discussed the potential importance of pattern recognition as a non-analytic clinical reasoning approach. However, this was explicitly raised by clinical teachers at UoM, for example as follows:
  “So, if you’re seeing a patient, you need some basic information on which you’re going to hang that, then you need to examine the patient, get the history and recognize that pattern, so that it can inform you. You need to recognise the pattern, which will then inform what further information you look for in terms of investigation or whatever.” (Clinical teacher 2, UoM)
  Clinical year students in both settings might be expected to observe the use of this non-analytical strategy, including intuition, by their clinical teachers. This was illustrated by 1 semester 12 student in UoM who was amazed by a professor who intuitively determined the need for further examination for a young woman with a urinary tract infection. According to this student, the professor just said “I don’t know why I ran the test, I just thought it wasn’t right” (FGD semester 12 UoM 2). UI students were far less likely to value intuitive thinking.

## Discussion

This study aimed to explore the influence of the culture of learning on clinical reasoning teaching and learning in 2 medical schools in Australia and Indonesia (UoM and UI), drawing on Hofstede’s dimensions. The use of the DTI demonstrated that CR proficiency might increase with medical training in both schools. Semester 12 students were more likely to be able to reflect on the items based on their experience in clinical settings compared to their semester 6 counterparts. The DTI, moreover, required the respondents to answer each item based on what they *would* do, not what they should do, in patient encounters [36]. These data were consistent with previous studies [37, 43, 44]. It should be noted, however, that the higher DTI scores only reflect a greater familiarity with the thinking process in diagnostic reasoning and do not necessarily represent better diagnostic performance.

Despite this, there were important differences found which appeared to have a cultural basis. Although clinical reasoning applies to both analytical and non-analytical approaches [15, 45], the Flexibility in Thinking subscale seemed to be inconsistent in the UI context. The DTI item analysis revealed 4 problematic items related to: early thought of diagnostic possibilities (Flexibility 2), clarification of symptoms (Flexibility 6), comparing and contrasting various possible diagnoses (Flexibility 13), and preparation of change of the decision about a patient (Flexibility 18). Another study using the DTI in Indonesia [44] confirmed this finding less consistency in the DTI Flexibility in Thinking subscale compared with the other subscale (Knowledge Structure in Memory). According to Bordage, the Flexibility in Thinking subscale measures ‘the use of variety in thinking processes’ during diagnostic processes (p. 415) [37], and it would seem important that the use of thinking processes should be flexibly adjusted to the nature of clinical problems encountered. This result appears to highlight a tendency toward uncertainty avoidance in the UI context, along with a preference for standardised CR processes. This was confirmed by multiple comments in the FGD data. The avoidance of risk and the need for standardised procedures are characteristics of strong uncertainty avoidance culture [22].

Secondly, the qualitative results of the present study suggest cultural differences in attitudes to learning clinical reasoning have the potential to impact on the learning process, especially in relation to 2 of Hofstede’s dimensions: differential attitudes to authoritative sources (power distance), and capacity to deal with uncertainty. These are highly relevant to medical diagnosis and the acquisition of new CR knowledge, given this process is developmental [46] and case specific [15, 16].

In a culture with a large power distance, as in the Indonesian context, teachers are mostly viewed as the definitive source of information and the learning process is highly influenced by them [22, 23], a fact borne out in the FGD data in this study. UI students appeared to rely on their authoritarian figures-teachers or tutors-for the provision of clear guidance and acquiring knowledge more than their UoM counterparts [25, 28]. This is consistent with Indonesian culture generally having a large power distance, with students showing dependence on authorities in the context of the higher education system [22]. This dependence had the potential to hinder students’ skills in constructing their knowledge and diagnostic scripts which are important for learning clinical reasoning skills [47]. Their views were in line with those of their teachers, who highly valued their dominant roles as content experts and seemed to adopt a comparatively authoritarian approach to teaching. It may also be the case that a more paternalistic culture of medical practice, as described by Hofstede et al. [21], exists within the UI setting. By contrast, UoM academics seemed consistently to position themselves as facilitators in their students’ learning process.

A second study in an Indonesian medical setting suggested the importance of acknowledging this power distance in providing feedback following Mini-CEX assessments in clinical settings [33]. The research showed better student outcomes following feedback when it was provided by senior clinicians rather than by residents. The view of UI students and academics regarding content authorities therefore needs to be considered in order to effectively facilitate students’ CR skills both in pre-clinical and clinical years. For example, the expertise of medical teachers would be expected to have a positive impact on students’ clinical reasoning when the thinking process is clearly verbalised [48]. However, since students also benefit from the opportunity to identify their weaknesses and reveal their difficulties in constructing biomedical and clinical knowledge necessary for CR and individual ‘illness script’ development [49, 50], it is important that they feel ‘safe’ in doing so. This would seem to require explicit opportunities and reassurance from teachers to enable this process.

Third, the tendency to seek complete information is a characteristic of a culture characterised by uncertainty avoidance [22]. The present study identified perceptions and practices consistent with uncertainty avoidance in the UI more so than in the UoM setting. Indeed, the curriculum at the UI emphasises information thoroughness; for example, history taking tutorials during pre-clinical years in particular is consistent with this cultural aspect. This is also consistent with the belief held by many Asian students that any information should be thoroughly committed to memory in order to be understood [51, 52]. However this preference may inhibit students’ clinical reasoning development, given that an effective process involves the capacity to select key information for hypothesis development, not just listing and memorising all information [53].

Finally, PBL was believed by UoM students and some academics to be an explicit way to learn clinical reasoning, especially in the pre-clinical training years. This was supported by consistent implementation of a progressive disclosure approach in the UoM PBL tutorials, where the data in the PBL trigger was given to students in a step-by-step manner according to the progress of the discussion. By contrast, despite the implementation of PBL at UI since 2005, most UI academics still felt PBL in the pre-clinical years should focus on content knowledge instead of giving equal importance to the facilitation of developing clinical reasoning skills. UI academics were particularly concerned that learning clinical reasoning too early could distract students’ attention from the acquisition of biomedical knowledge. This was a fundamental contrast with western approaches to the use of PBL in medical education [54, 55].

A characteristic of uncertainty avoidance was also suggested among UI academics who did not suggest pattern recognition as a strategy that can be learned by medical students. A pattern recognition strategy is largely based on the physician’s experience and proceeds more automatically than the analytic approach [45, 56]. Given the context specificity of the CR process [15, 16], including the more automatic process of pattern recognition, it might be hard to standardise this for teaching purposes, and this may explain to some extent the goal of providing a structured approach by emphasising analytical clinical reasoning to medical students at the UI.

The issues are potentially significant given previous studies by Jippes and Majoor have suggested that greater power distance and avoidance of uncertainty are negatively correlated with the implementation of an integrated curricula and PBL in select global medical schools [31, 32]. This study confirms these conclusions by identifying attitudes to authority and uncertainty avoidance as possible additional influences on clinical reasoning teaching and learning.

### Limitations of the study

This comparative case study research was based on a modestly sized and specific population sample, which obviously limits the generalisability of the research findings [34, 35]. It captured the differences in attitudes to clinical reasoning of semester 6 and 12 students using the DTI in the 2 settings, noting we did not perform a cohort study to assess the attitude change or development. Despite this limitation, we confirmed the findings from previous studies for different levels of students undertaking comparable curricula [37, 42, 44].

Furthermore, this study did not attempt to assess actual clinical reasoning performance as evidence of the impact of culture on the effectiveness of students’ clinical reasoning. Further study would be required to address this. The systematic differences pertaining to the influence of power distance and uncertainty avoidance addressed in this study however appear to be important for clinical reasoning teaching and learning.

## Conclusion

A vast literature exists on the nature of medical education in western settings. By contrast there are few publications to date (in English or other languages) on the nature of medical education in Asian institutions, despite the growth in medical schools in Asia and Africa in the past 30 years [1, 2].

This study explored possible influences of culture of learning and the adoption of western curricula or methods in Asian medical schools. It did not attempt to predict whether the differences in culture of learning would influence the capacity of clinical reasoning of the graduates at the end of the course. However, given the tendencies of adoption of western methods worldwide, including in countries with different cultures of learning, this study highlights the importance of considering local cultures of learning when adopting western approaches.

The Diagnostic Thinking Inventory (DTI) results suggest that attitudes pertaining to the structure of memory and flexibility in thinking of the more advanced students (semester 12) score more highly than those of students in the earlier stages of training (semester 6). This may show potential development of attitudes towards a more advanced level of training which would require more detailed investigation in a future cohort study. This study also demonstrates the potential influence of culture on clinical reasoning teaching and learning. It suggests that cultural differences in attitudes to authoritative sources and attitudes to uncertainty between the UoM and the UI influence clinical reasoning conceptualisation as well as certain aspects of teaching and learning clinical reasoning in each setting. The culture of learning in each medical school and the understanding of the nature of clinical reasoning expertise development can guide medical teachers and curriculum developers in creating the most effective teaching and learning activities. In order to implement teaching and learning strategies for clinical reasoning, particular cultural issues such as those related to power distance and uncertainty avoidance tendency need to be understood and addressed. Given the current growth of medical schools in the Asian region, these issues are particularly important.
